# Nogo-A-targeting antibody promotes visual recovery and inhibits neuroinflammation after retinal injury

**DOI:** 10.1038/s41419-020-2302-x

**Published:** 2020-02-06

**Authors:** Julius Baya Mdzomba, Sandrine Joly, Léa Rodriguez, Ali Dirani, Patricia Lassiaz, Francine Behar-Cohen, Vincent Pernet

**Affiliations:** 1 CUO-Recherche, Centre de recherche du CHU de Québec-Université Laval and Département d’ophtalmologie, Faculté de médecine, Université Laval, Québec, Québec, Canada; 2 Inserm UMR_S 1138, Team 17, Centre de Recherche des Cordeliers, Paris Descartes University, Sorbonne University, and University of Pierre et Marie Curie, Paris, France

**Keywords:** Retina, Neurodegeneration

## Abstract

N-Methyl-D-aspartate (NMDA)-induced neuronal cell death is involved in a large spectrum of diseases affecting the brain and the retina such as Alzheimer’s disease and diabetic retinopathy. Associated neurological impairments may result from the inhibition of neuronal plasticity by Nogo-A. The objective of the current study was to determine the contribution of Nogo-A to NMDA excitotoxicity in the mouse retina. We observed that Nogo-A is upregulated in the mouse vitreous during NMDA-induced inflammation. Intraocular injection of a function-blocking antibody specific to Nogo-A (11C7) was carried out 2 days after NMDA-induced injury. This treatment significantly enhanced visual function recovery in injured animals. Strikingly, the expression of potent pro-inflammatory molecules was downregulated by 11C7, among which TNFα was the most durably decreased cytokine in microglia/macrophages. Additional analyses suggest that TNFα downregulation may stem from cofilin inactivation in microglia/macrophages. 11C7 also limited gliosis presumably via P.Stat3 downregulation. Diabetic retinopathy was associated with increased levels of Nogo-A in the eyes of donors. In summary, our results reveal that Nogo-A-targeting antibody can stimulate visual recovery after retinal injury and that Nogo-A is a potent modulator of excitotoxicity-induced neuroinflammation. These data may be used to design treatments against inflammatory eye diseases.

## Introduction

Excitotoxic cell death mediated by N-methyl-D-aspartate (NMDA) receptor activation plays a central role in the neurodegenerative diseases affecting the CNS, including the retina^1–4^. In retinal diseases such as diabetic retinopathy, irreversible visual deficits have been linked to NMDA receptor overactivation^5–7^ that results in the death of retinal ganglion cells (RGCs), the output neurons of the retina that normally ensure the transfer of visual information from the eye to the brain^8,9^. The toxic levels of glutamate in diabetic retinal^7^ and vitreous^5^ samples are due to the defects in glutamate detoxification^7^ and reuptake^10^ by glial cells. Consistently, intravitreal injections of NMDA in rodents is a classical model to study the mechanisms of excitotoxity in the retina^8,11–13^.

In the present study, we wondered if Nogo-A influenced the development of excitotoxic damage in the retina. Nogo-A is a potent myelin-associated inhibitor for neuronal plasticity and axonal regeneration in the injured CNS^14,15^. At the molecular level, three distinct inhibitory domains of Nogo-A have been identified^16^. In its N-term part, Nogo-A contains an amino acid sequence inhibiting fibroblast cell spreading. A specific central region of the protein called Nogo-A-Delta 20 restricts cell spreading and neurite outgrowth and a C-term domain called Nogo-66 promotes growth cone collapse. The cell transduction mechanisms of Nogo-A depend on the interaction between its three domains and different receptor partners. Nogo-66 binds and activates a receptor complex composed of Nogo-66 receptor 1 (NgR1)^17^, Lingo-1^18^ and p75/Troy^19,20^ leading to actin cytoskeleton disassembly via the small GTPase RhoA^21^. On the other hand, Nogo-A-Delta 20 can be a ligand for sphingosine 1-phosphate receptors (S1PR)^22^. Its binding to S1PR2 intracellularly activates the RhoA-ROCK pathway in fibroblasts and neuronal cells. Targeting Nogo-A-Delta 20 with neutralizing antibodies such as 11C7 was shown to promote neurological recovery in experimental models of stroke^23–25^ and spinal cord injury^26,27^.

The involvement of Nogo-A in pathological mechanisms affecting the retina suggests that Nogo-A-targeting antibodies may also improve vision in ocular diseases. Our recent results revealed that blocking Nogo-A-Delta 20 with intravitreal injection of 11C7 antibody dramatically increased vascular repair and neuronal function in the retina of mice subjected to ischemic retinopathy^28^. In adult mice, we also reported that Nogo-A KO mice had better visual recovery after retinal injury than WT animals^29^. Here, we hypothesized that retinal Nogo-A may inhibit injured neuron repair^28,30–33^. Indeed, immunofluorescent observations revealed high levels of Nogo-A in radial Müller glia, a specialized retinal glia, and to a lower extent in RGCs.

In this study, retinal injury caused the simultaneous release of Nogo-A and TNFα in the mouse vitreous. Neutralizing Nogo-A-Delta 20 with intravitreal injection of 11C7 improved visual function recovery and strongly reduced inflammation at the same time. Importantly, Nogo-A was dramatically increased in the retina and in the vitreous humor of donors suffering from diabetic retinopathy. Taken together, these results unveil a new function of Nogo-A in retinal inflammation.

## Materials and methods

### Animals

In this study, adult male C57BL/6J mice were purchased from the Jackson Laboratory and were used at 2–4 months of age. All animal experiments were carried out in accordance with the guidelines of the Canadian Council on Animal Care and of the Animal Welfare Committee of the Université Laval.

### Intraocular injections

To induce retinal injury, 2 µl of NMDA (0.5–40 nmol) were injected in the left eye using a 10-µl Hamilton syringe, under general anesthesia with isoflurane, as previously described^29,32–35^. In brief, the glass tipped needle was delicately inserted through the sclera of the eyeball by taking care not to injure the lens. To allow NMDA diffusion in the vitreous and limit its backflow, the needle was held in place for 3 min and then slowly withdrawn. A drop of surgical glue (Histoacryl®, Braun) was applied to seal the injection site. The same procedure was used for the injection of the function-blocking antibody 11C7 (IgG1 serotype, 2 μg/eye, a gift from Prof Martin Schwab, Univ Zurich,), raised against Nogo-A-Delta 20^16^, or control IgG (IgG1 serotype, clone FG12/B5, 2 μg/eye, Antibody Production Service Ltd)^29^, 2 days after NMDA injection.

### Optokinetic reflex test

To evaluate the visual acuity of freely moving mice, the optokinetic reflex was tested using OptoDrum system (Striatech GmbH Vor dem Kreuzberg, Tübingen, Germany). In brief, mice (6–9/group) were placed on a platform in the middle of an arena surrounded by four computer screens while moving gratings of different spatial frequencies were passed on the monitors^36,37^. The reflexive tracking movement of the mouse neck in the temporal-to-nasal direction allowed to evaluate the visual response of the two eyes separately by changing the direction of the visual stimulus. For statistical analysis, a two-way ANOVA was applied (GraphPad Prism, GraphPad Software, La Jolla, CA, USA).

### Electroretinogram (ERG) recording

A Ganzfeld system (Phoenix Research Labs, Pleasanton, CA, USA) was used to record photopic ERGs in mice (5–9/group) anesthetized with a ketamine/xylazine mixture, as described before^28,38^. Prior to recording, a drop of 1% Mydriacyl Tropicamide was applied on the cornea for pupil dilation. A sterile ophthalmic gel (Tear-Gel, Baush & Lomb) was used to prevent corneal desiccation and to allow the contact between the cornea and the electrode (gold-plate objective lens). ERGs were generated in response to flash stimulations of increasing intensities, ranging from 1.0 log cd.s.m^2^ to 2.8 log cd.s.m^2^ (inter-stimulus interval, 20 ms; flash duration, 1 ms; average of 20 flashes, 0.6 log-unit increment). The use of green (504 nm) and UV (365 nm) lights allowed to record M-cone and the S-cone-dependent ERG waveforms respectively. The amplitude of the b-wave was measured from the a-wave trough to the highest peak. Statistical analysis was performed using a two-way ANOVA followed by Tukey post hoc test (GraphPad Prism).

### Tissue preparation for histological analysis

Mice were euthanized using a lethal dose of ketamine/xylazine mixture (90–10 mg/kg). Tissues were fixed by intracardial perfusion of phosphate-buffered saline (PBS) and 4-% paraformaldehyde (PFA) solutions. Eyes and optic nerves were dissected for retinal and optic nerve sections (14-µm thick), or retinal flat-mount preparation.

### Retinal ganglion cell (RGC) and amacrine cell survival

The survival of RGCs and amacrine cells was evaluated 43 days after NMDA injection on retinal flat-mounts of 6–9 mice for each experimental group. Retinal flat-mounts were post-fixed overnight in 4% PFA, rinsed with PBS three times, incubated for 1 h in a blocking solution (PBS containing 5% bovine serum albumin (BSA), 0.3% Triton X-100, and 0.01% sodium azide) and incubated for 5 days at room temperature with the following primary antibodies: rabbit anti-RNA binding protein with multiple splicing (RBPMS) (1:200, PhosphoSolutions, Aurora, CO, USA, #1830-RBPMS) or goat anti-choline acetyltransferase (ChAT) (1:100, Millipore, #AB144P). After intensive washing, retinal flat-mounts were incubated for 2 days at room temperature with appropriate secondary antibodies diluted in the blocking solution. Vectashield solution (BioLynx, Brockville, ON, Canada) was used as a mounting medium. Cells were counted in regions of 62,500 µm^2^ at 0.5, 1, 1.5, and 2 mm from the optic disk in the four retinal quadrants. Statistical differences were evaluated using an unpaired *t*-test (GraphPad Prism).

### Immunofluorescence on retinal and optic nerve cryosections

Retinal eye cups and optic nerves were post-fixed overnight in 4% PFA and immersed in a solution of 30% of sucrose for cryo-protection before tissue embedding in optimal cutting temperature (Cedarlane, Burlington, ON, Canada) medium. 14-µm thick cryosections were collected on Superfrost microscope glass slides. For immunostaining, tissue slices were incubated for 1 h in a blocking solution and then overnight with primary antibodies at 4 °C (see Table 1). After three washes with PBS sections were incubated at room temperature with the appropriate secondary antibodies. Slides were mounted with the Vectashield mounting medium. For microscopy and image acquisition, mosaic pictures were taken with a Zeiss AxioImager M2 microscope equipped with a motorized platform and the ZEN software and a LSM 700 scanning confocal microscope (Zeiss). All quantifications were performed using 5–6 central retinal or optic nerve cuts/mouse. At least 3 mice were used per experimental condition. In quantitative analyses, statistical significance was evaluated using a one-way ANOVA followed by Dunnett’s post hoc test.

**Table 1:** Antibodies used for immunofluorescence (IF) and western blotting (WB).

Name	Species	Dilution IF	Dilution WB	Source	Catalog #
RBPMS	Guinea pig	1:200		PhosphoSolutions (Aurora, CO)	1832-RBPMS
RBPMS	Rabbit	1:200		PhosphoSolutions (Aurora, CO)	1830-RBPMS
P.Stat3	Rabbit	1:100	1:500	Cell Signaling	9131
Stat3	Rabbit		1:1 000	Cell Signaling	D3Z2G
P.cofilin	Rabbit	1:200	1:1 000	Cell Signaling	3313
Cofilin	Rabbit		1:1 000	Cell Signaling	5175
Iba-1	Rabbit	1:200		Wako	019-19741
Iba-1	Goat	1:1 000		Novus Biologicals	NB100-1028
Isolectin b4		1:100		Life Technologies	I21413
GFAP	Rabbit	1:500		Dako	Z-0334
Glutamine synthetase	mouse	1: 500		Millipore	MAB302
P.Erk	Rabbit	1:100	1:1 000	Cell Signaling	4370
Erk	Rabbit		1:1 000	Cell Signaling	4595
TNFα	Mouse	1:200		Abcam	ab1793
TNFα	Rabbit		1:1000	Abcam	ab2148P
CD68	Rat	1:1 000		Abcam	ab53444
Nogo-A	Rabbit		1:1 000	Abcam	Ab47085
Nogo-A (Rtn4)	Rabbit	1:200	1:2 000	Dr. M. Schwab	Rb173A (Laura)
Nogo-A	Rabbit		1:20 000	Dr. M. Schwab	Rb1 (Bianca)
Nogo-A	Sheep		1:5 000	Dr. M. Schwab	S544
Nogo-A	Mouse	1:200	1:2 000	Dr. M. Schwab	11C7
Olig2	Rabbit	1:500		Millipore	AB9610
Smi-32	Mouse	1:500		Millipore	NE1023
GAPDH	Mouse		1:20 000	Abcam	ab8245

### Western blot analysis

Retinae were quickly isolated from the eyes, snap frozen in liquid nitrogen and stored at −80 °C until protein lysate preparation. Retinae were homogenized for 60 min on ice in Eppendorf tubes containing lysis buffer (20 mM Tris-HCl, 0.5% CHAPS, pH 8.0) and protease/phosphatase inhibitor (Roche Diagnostics, Laval, QC, Canada) and centrifuged for 15 min at 15,000 × *g* at 4 °C. Supernatants were then retrieved and used for protein assay (BioRad, Mississauga, ON, Canada). Retinal proteins (20 μg/well) were resolved by electrophoresis on 4–12% gradient polyacrylamide gels and transferred to nitrocellulose membranes. Nitrocellulose membranes were pre-incubated in a blocking solution of 5% BSA dissolved in TBST (Tris-base 0.1 M, 0.2% Tween 20, pH 7.4) for 1 h at room temperature, incubated with primary antibodies overnight at 4 °C (see Table 1). After washes, membranes were incubated with a horseradish peroxidase-conjugated anti-mouse or anti-rabbit antibody (1:10 000; Pierce Biotechnology, Burlington, ON, Canada). Chemiluminescent bands were detected with LiCor Western Sure Premium Chemiluminescent Substrate (Mandel, Guelph, ON, Canada) in a LiCor C-Digit blot scanner (Mandel). Band signals were quantified with the ImageJ software and analyzed with the GraphPad Prism software. To study the dose-dependent effects of NMDA on retinal protein expression changes, 3 mice were used for each experimental condition. In this case, statistics were done using a one-way ANOVA followed by Dunnett’s post hoc test (GraphPad Prism). The effects of 11C7 and control IgG were analyzed in retinal and vitreous lysates using 3–5 mice per group, as indicated in the figure legends. Statistical analysis was performed using an unpaired *t*-test (GraphPad Prism).

### Semi-qRT-PCR

Animals were sacrificed by cervical dislocation and tissue rapidly obtained before being flash-frozen in liquid nitrogen. Eppendorf tubes of the tissue collected were stored at −80 °C until RNA could be extracted. To prepare total retinal RNA, an RNeasy isolation kit (Qiagen, Toronto, ON, Canada) was used. Residual genomic DNA was eliminated by use of a DNase treatment. Oligo (dt) and M-MLV reverse transcriptase (Fisher Scientific) was used to transform equal amounts of RNA for reverse transcription. Amplification of ten nanograms of cDNA with the SYBR Green I Master polymerase ready mix (Roche Diagnostics Canada) was done using the Light Cycler 480 thermocycler (Roche Diagnostics Canada). Primer pairs were made to span the intronic sequences or to cover exon-intron boundaries (see Table 2 for sequences). The comparative threshold cycle (ΔΔCT) method was used to calculate the relative quantification. As a reference gene and control sample, *Gapdh* was used to normalize cDNA levels. Each reaction was done in triplicate for 4–6 mice per condition. Statistical analysis was performed by applying an unpaired *t*-test (GraphPad Prism).

**Table 2:** Primer sequences used for qRT-PCR measurements.

Gene Names	Sequence forward(5’-3’)	Sequence reverse (5’-3’)	Product (bp)
*Aif1*	GGAGACGTTCAGCTACTCTGAC	GCCCTGATTGGAGGTGGATG	163
*Atf3*	ACCTCCTGGGTCACTGGTATTTG	TTCTTTCTCGCCGCCTCCTTTTCC	215
*Bdnf*	CAAAGCCACAATGTTCCACCAG	GATGTCGTCGTCAGACCTCTCG	213
*Ccl2*	GGCTCAGCCAGATGCAGTTA	CTGCTGCTGGTGATCCTCTT	108
*CD68*	ACCTACATCAGAGCCCGAGTACAG	TTCTGCGCCATGAATGTCCACTG	100
*Cntf*	CTCTGTAGCCGCTCTATCTG	GGTACACCATCCACTGAGTC	125
*Cox2*	GACAGATCATAAGCGAGGAC	TACACCTCTCCACCAATGAC	153
*Csf1*	GCTCCAGGAACTCTCCAATA	TCTTGATCTTCTCCAGCAGC	119
*Cx3cl1*	CCGCGTTCTTCCATTTGT	CTGTGCTGTGTCGTCTCCA	175
*F4/80*	TGGGACAAACACTTGGTGGTGTG	GTGTCAGTGCAGGTGGCATAAG	76
*Gapdh*	CAGCAATGCATCCTGCACC	TGGACTGTGGTCATGAGCCC	96
*Gap43*	TGCTGTCACTGATGCTGCT	GGCTTCGTCTACAGCGTCTT	127
*Gfap*	CCACCAAACTGGCTGATGTCTAC	TTCTCTCCAAATCCACACGAGC	240
*Il-1b*	GCTATGGCAACTGTTCCTGA	GATGTGCTGCTGCGAGATT	171
*Lif*	AATGCCACCTGTGCCATACG	CAACTTGGTCTTCTCTGTCCCG	216
*Rtn4*	CAGTGGATGAGACCCTTTTTG	GCTGCTCCTTCAAATCCATAA	90
*Rtn4r*	CTCGACCCCGAAGATGAAG	TGTAGCACACACAAGCACCAG	116
*S1pr1*	TCAGGGAACTTTGCGAGTGA	AACAGCAGCCTCGCTCAAG	123
*S1pr2*	CATCGCCATCGAGAGACAAG	TCAGACAATTCCAGCCCAGG	146
*Sprr1a*	GAACCTGCTCTTCTCTGAGT	AGCTGAGGAGGTACAGTG	91
*Sphk1*	ATACTCACCGAACGGAAGAAC	ATTAGCCCATTCACCACCTC	122
*Sphk2*	GCTTTACGAGGTGCTGAATG	AGAAGCGAGCAGTTGAG	174
*Stat3*	CAAAACCCTCAAGAGCCAAGG	TCACTCACAATGCTTCTCCGC	139
*Tnf*	CCACGCTCTTCTGTCTACTGA	GGCCATAGAACTGATGAGAGG	92
*Tubb3*	GGCCTCCTCTCACAAGTATG	TTGCCAGCACCACTCTGAC	138
*Vim*	TACAGGAAGCTGCTGGAAGG	TGGGTGTCAACCAGAGGAA	113

### Human retina histology and vitreous analyses

All patients involved in the study signed an informed consent prior to vitreous or retinal use. Experiments with human samples have been approved by the Comité d’éthique de la recherche du CHU de Québec-Université Laval. For immunofluorescence observations, human retinal sections were obtained from a french eye bank association (Banque française des yeux, Paris, France). Paraffin sections were cut from the eye of a non-diabetic, 71-year-old male donor. The cause of the death was renal insufficiency. Diabetic retinal sections came from a 85-year old male donor suffering from type 2 diabetes, treated with insulin and presenting non-proliferative diabetic retinopathy with macular edema. The origin of the death was lung carcinoma. For the two donors, retinae where collected up to 20 h post-mortem. Retinal cross sections were stained for glutamine synthetase (GS antibody, 1:300, MAB 302, Chemicon Technology, Merck Millipore, Billerica, Massachusetts, USA), for glial fibrillary acidic protein (GFAP antibody, 1:300, Abcam, ab49874, Cambridge, UK) and Nogo-A (11C7 antibody, 1:200). For the detection of Nogo-A in the human vitreous, vitreous samples were collected by an experienced ophthalmologist of the Hôpital du Saint-Sacrement of the Centre Universitaire d’ophtalmologie in Quebec City, Canada. Analyzed samples were obtained from two non-diabetic male patients of 73 and 75 years of age treated for retinal detachment and epiretinal membrane, respectively. Vitreous of diabetic patients were obtained from a 77-year-old man, without diabetic retinopathy, and from a 68-year-old woman with proliferative diabetic retinopathy. To monitor the protein level of Nogo-A, fifteen microliters of vitreous was analyzed by western blot using 11C7 antibody (1:2000). The ability of 11C7 to detect human Nogo-A was validated using recombinant Nogo-A protein (Abcam, ab163499).

## Results

### Retinal excitotoxicity increases the level of Nogo-A proteins in the vitreous of mice

**Fig. 1:**
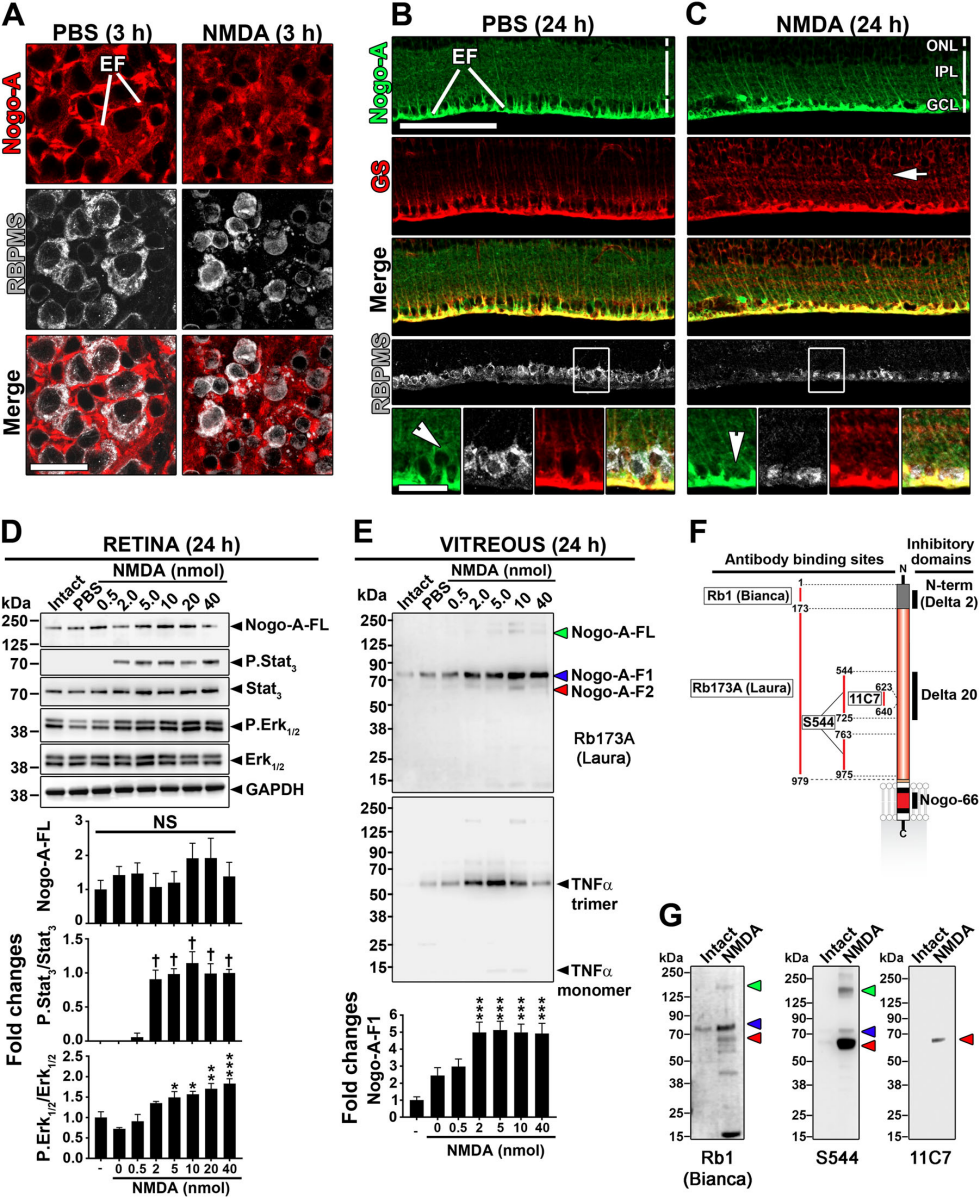
Retinal injury induces Nogo-A protein release in the mouse vitreous. **a**–**c** Compared with intact condition (no treatment) and PBS treatment, intravitreal injection of NMDA induced the loss of retinal ganglion cells expressing Nogo-A and the specific cell marker RBPMS. In contrast, glial expression of Nogo-A did not change in glutamine synthetase (GS)-expressing Müller cells after NMDA-induced injury. Three mice per condition were examined. **d** By Western Blotting, the level of Nogo-A protein did not significantly change in retinal lysates treated with increasing doses of NMDA (0–40 nmoles) although P.Stat_3_ and P.Erk_1/2_ were upregulated in a dose-dependent manner in response to retinal damage. **e** Western Blot analysis of mouse vitreous (4 μL/well) revealed that NMDA induced the increase of Nogo-A and TNFα proteins in a dose-dependent manner. For quantitative analysis, 3 mice were used per condition. With Rb173A antibody which recognizes the Nogo-A specific domain encoded by exon 3, 4 proteins were observed at ~80 kDa (Nogo-A-F1), ~68 kDa (Nogo-A-F2), and at ~200–250 kDa (Full-length Nogo-A, Nogo-A-FL). Strikingly, TNFα appeared predominantly in trimers, i.e. under its most inflammatory form. **f**, **g** Additional antibodies directed against different parts of Nogo-A, named Rb1, S544, and 11C7, were used to determine the presence of Nogo-A proteins in the vitreous of NMDA-treated eyes. Increased levels of Nogo-A proteins were thus confirmed. Interestingly, proteins of similar molecular weight were found and may correspond to Nogo-A-F1, Nogo-A-F2, and Nogo-A-FL. Statistics: One-way ANOVA, Dunnett’s post hoc test, *P < 0.05; **P < 0.01; ***P < 0.001; ^†^*P* < 0.0001. Scale bars: **a**, **b** close-up (bottom left) = 25 μm; **b** (top left) = 100 μm.

Nogo-A is endogenously expressed in Müller glia and ganglion cell (RGC) bodies^30,32,33^. By immunofluorescence and western blotting (Fig. 1), we set out to determine if NMDA-induced neuronal cell death was associated with changes in Nogo-A localization and expression. Three hours after NMDA intravitreal injection (2 nmol/eye), RGC lysis was established by the observation of fragments labeled with *RNA-binding protein with multiple splicing* (RBPMS), a protein selectively localized in the soma of RGCs^39^, suggesting quick extracellular spill of RGC cytoplasm after excitotoxicity induction (Fig. 1a). On retinal cross sections, 24 h after NMDA-induced injury, many large soma-sized RGCs expressing RBPMS and Nogo-A were lost (Fig. 1b, c). In NMDA-treated animals, remaining RGCs displayed a weak signal for Nogo-A (Fig. 1b, c, arrow). However, Nogo-A fluorescent signal was not much different in Müller cell processes labeled with glutamine synthetase (GS), compared with that observed in intact or PBS-injected retinae. To further investigate the possible release of Nogo-A following NMDA-induced RGC lysis, a Western blot analysis of retinal and vitreal lysates was undertaken (Fig. 1d–g). The level of Nogo-A protein did not significantly change after the injection of increasing doses of NMDA in the vitreous humor (Fig. 1d). The phosphorylation of Stat3 and Erk1/2 has been shown to participate in the neuronal and glial response to retinal injury^12,40–42^ and was thus used to monitor the reaction of retinal cells to NMDA injury. We observed that P.Stat3 and P.Erk1/2 were upregulated by NMDA in a dose-dependent manner (Fig. 1d). In contrast, the lack of change for Nogo-A (Fig. 1d) is attributable to stable expression in Müller glia, i.e. the cells constituting the main source of this protein in the whole retina^30–33^. Strikingly, however, Nogo-A proteins dose-dependently increased in the vitreous of eyes treated with NMDA (Fig. 1e). The Nogo-A proteins observed in the vitreous were full-length Nogo-A and protein fragments that were named Nogo-A-F1 and Nogo-A-F2. Similar Nogo-A proteins could be detected in the vitreous of NMDA-injected eyes using antibodies recognizing its three inhibitory domains (Fig. 1f, g). Coincidental increase of the TNFα cytokine level suggests a link between retinal inflammation and Nogo-A elevation in the vitreous. Interestingly, Nogo-A could not be detected in the vitreous of mice subjected to the model of optic nerve injury (data not shown), a lesion paradigm whose damage exclusively depends on apoptosis^43^. In this process, apoptotic cell bodies are phagocytosed without cytoplasmic spillover in surrounding tissues. Taken together, these results suggest that Nogo-A proteins are released by RGCs in the vitreous after excitotoxic shock.

### Targeting Nogo-A-Delta 20 with a function-blocking antibody improves visual recovery after retinal injury

**Fig. 2:**
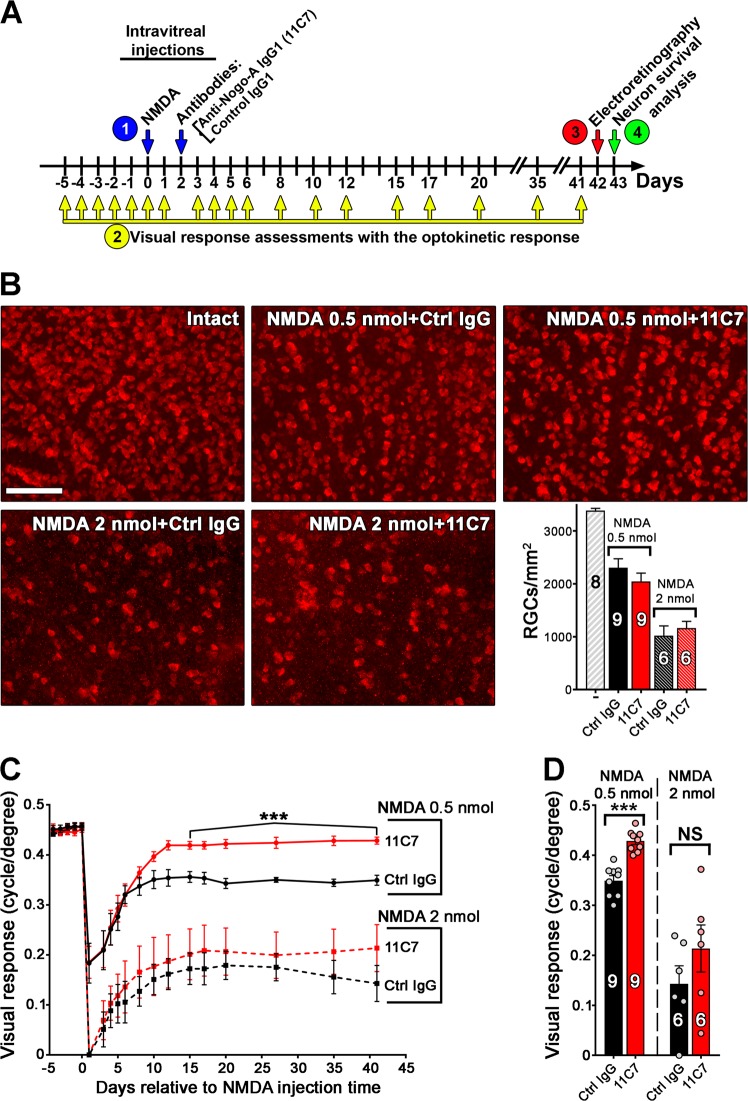
Intravitreal administration of Nogo-A blocking antibody enhances visual recovery after retinal injury. **a** To follow visual function changes following NMDA-induced injury (1), the optokinetic response was monitored before and after intravitreal injections (2). Control IgG (2μg/eye) or anti-Nogo-A IgG (11C7, 2 μg/eye) was delivered in the vitreous 2 days after NMDA injection. Electroretinograms were recorded in photopic conditions 6 weeks after NMDA injections (3, see Fig. S1). Retinal ganglion cell survival was assessed on retinal flat-mounts stained for RBPMS, the day following ERG recordings (4). At 0.5 nmol of NMDA, nine mice were examined for each antibody treatment (control IgG or 11C7). Six mice received control IgG and 11C7 after the injection of 2 nmol NMDA. **b** Immunofluorescent staining with RBPMS antibody revealed a ~30% reduction in the density of RGCs after injection of 0.5 nmol of NMDA and a ~70-% reduction following the administration of 2 nmol of NMDA relative to intact retinae. The level of cell death did not statistically vary between the two groups receiving either 11C7 or control IgG. **c** The optokinetic response of mice receiving 0.5 nmol of NMDA showed significant function deficits after control IgG treatment. Similar visual loss was obtained with 0.5 nmol NMDA alone, without antibody (data not shown). In contrast, blocking Nogo-A with 11C7 allowed much better recovery of optokinetic response sensitivity. The effect of 11C7 was not significant when injected after 2 nmol of NMDA. **d** The optokinetic response of individual animals revealed weak variability in groups injected with 0.5 nmol of NMDA compared with 2 nmol of NMDA. Statistics: **c**, ***P < 0.001, two-way ANOVA; **d**, ***P < 0.001, unpaired *t*-test. Scale bar = 100 μm.

The detection of Nogo-A-Delta 20 in the vitreous of injured eyes suggests that it may contribute to visual function loss after injury. This region of Nogo-A strongly inhibits neuronal repair/plasticity in the CNS^44^ but can efficiently be neutralized in vivo using 11C7 antibody^26–29,45^. We therefore tested the function of Nogo-A-Delta 20 in NMDA-induced visual deficits by injecting 11C7 (2 μg/eye) or an equal amount of control IgG in the mouse vitreous (Fig. 2a). In order to assess the therapeutic potential of anti-Nogo-A-Delta 20 antibody in retinal injuries, our experimental design consisted of performing the injection of 11C7 two days after that of NMDA (Fig. 2a). Given the fast kinetic of RGC cell elimination that is usually observed after NMDA injection^12,13,46^, a 2-day delay between antibody and NMDA treatment is relatively long and is thus expected to minimize the influence of 11C7 on RGC survival. The optokinetic response was evaluated repeatedly before and after injury to monitor visual function variations^29,40^. Electroretinogram recordings allowed to follow retinal cell activity upstream of RGCs^28,38^. A quantitative analysis of surviving RGCs was carried out on retinal flat-mounts stained for RBPMS (Fig. 2b). In these conditions, intraocular injection of 0.5 nmol and 2 nmol of NMDA gave rise to moderate (~30%) and massive (~70%) elimination of RGCs (Fig. 2b). The magnitude of RGC death obtained with 0.5 nmol of NMDA in our study is similar to that observed after prolonged ocular hypertension in mouse glaucoma models, as reported by others^47^. As anticipated, 11C7 did not change the density of surviving RGCs at either dose of NMDA (Fig. 2b). Similarly, the death of cholinergic amacrine cells was not affected by 11C7 in the inner nuclear layer and in the ganglion cell layer (Data not shown). However, visual function was significantly increased by 11C7 treatment after the injection of 0.5 nmol of NMDA (Fig. 2c, d). After 2 nmol of NMDA, only a trend towards an improvement could be noticed, probably due to massive neuronal cell death (Fig. 2c, d). Moreover, 11C7 did not affect ERG wave amplitudes, suggesting that it is not protective for the retinal activity generated by cells upstream of RGCs (Fig. S1). Together, these results show that inhibiting Nogo-A-Delta 20 with a single injection of 11C7 is sufficient to increase visual recovery without affecting neuronal survival.

### Nogo-A antibody inhibits monocyte-mediated inflammation in the injured retina

**Fig. 3:**
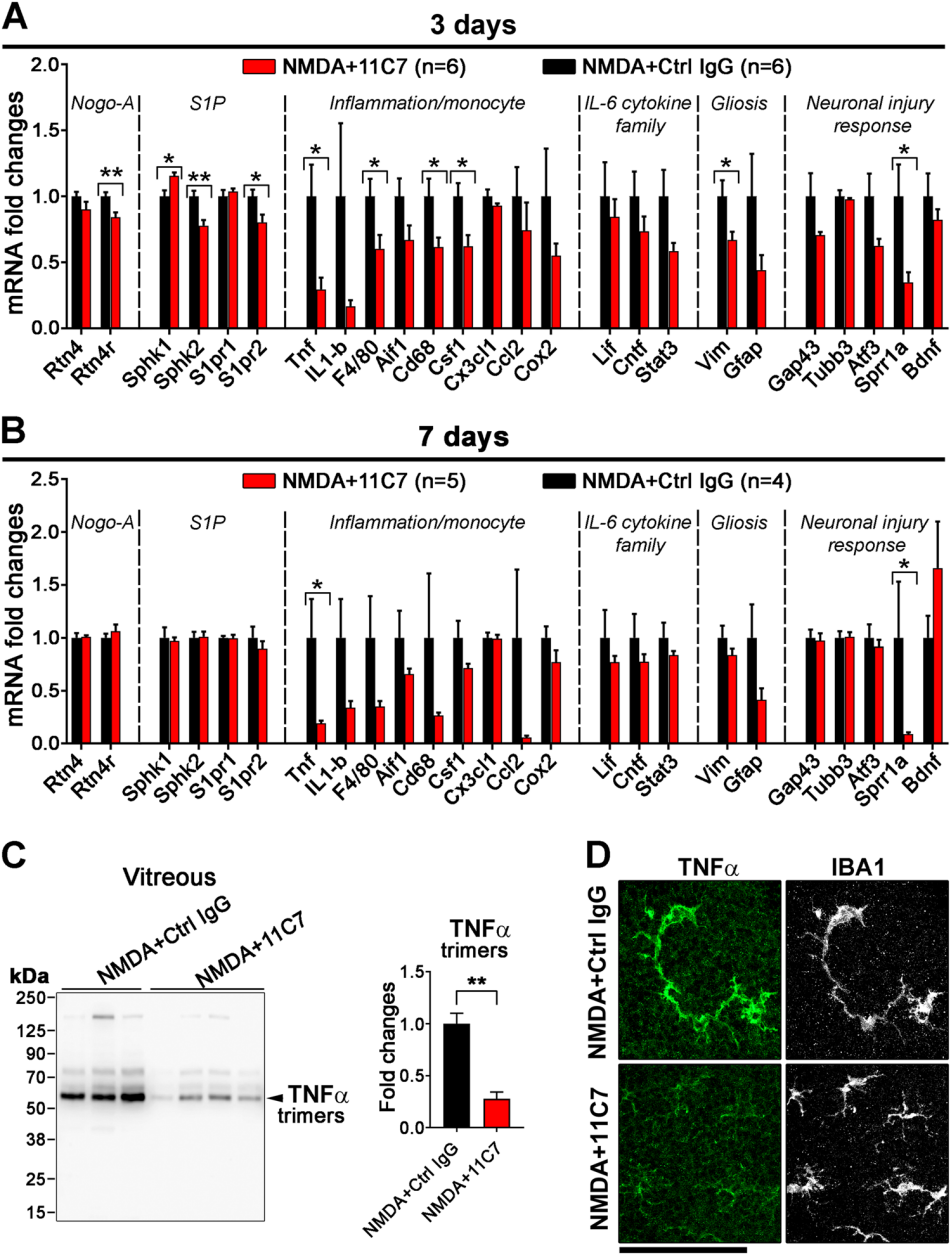
Antibody-based neutralization of Nogo-A mitigates inflammation in the injured retina. **a**, **b** Retinal gene expression analysis by qRT-PCR at 3 and 7 days after NMDA injection (0.5 nmol) and antibody treatments (2 μg/eye). Four-six mice were used per condition. 11C7 reduced the expression of major genes involved in inflammation and monocyte activation (*P < 0.05; **P < 0.01; unpaired *t*-test). In particular, 11C7 injection led to sustained decrease of *Tnf* transcript and *Sprr1a* mRNA, the expression of which reflects the severity of neuronal injury response. **c** The level of TNFα trimers was studied by Western Blotting the day after antibody injection. Compared with control IgG (*n* = 3 mice), 11C7 injections (*n* = 4 mice) significantly decreased the content of TNFα in the vitreous of injured eyes (**P < 0.01, Unpaired *t*-test). **d** TNFα was mainly observed in Iba1-expressing monocytes by immunofluorescence on retinal flat-mounts. Its signal was much weaker with 11C7 than in control mice. Immunofluorescent staining observations were repeated in three different mice for each antibody treatment. Scale bar in **d** = 100 μm.

**Fig. 4:**
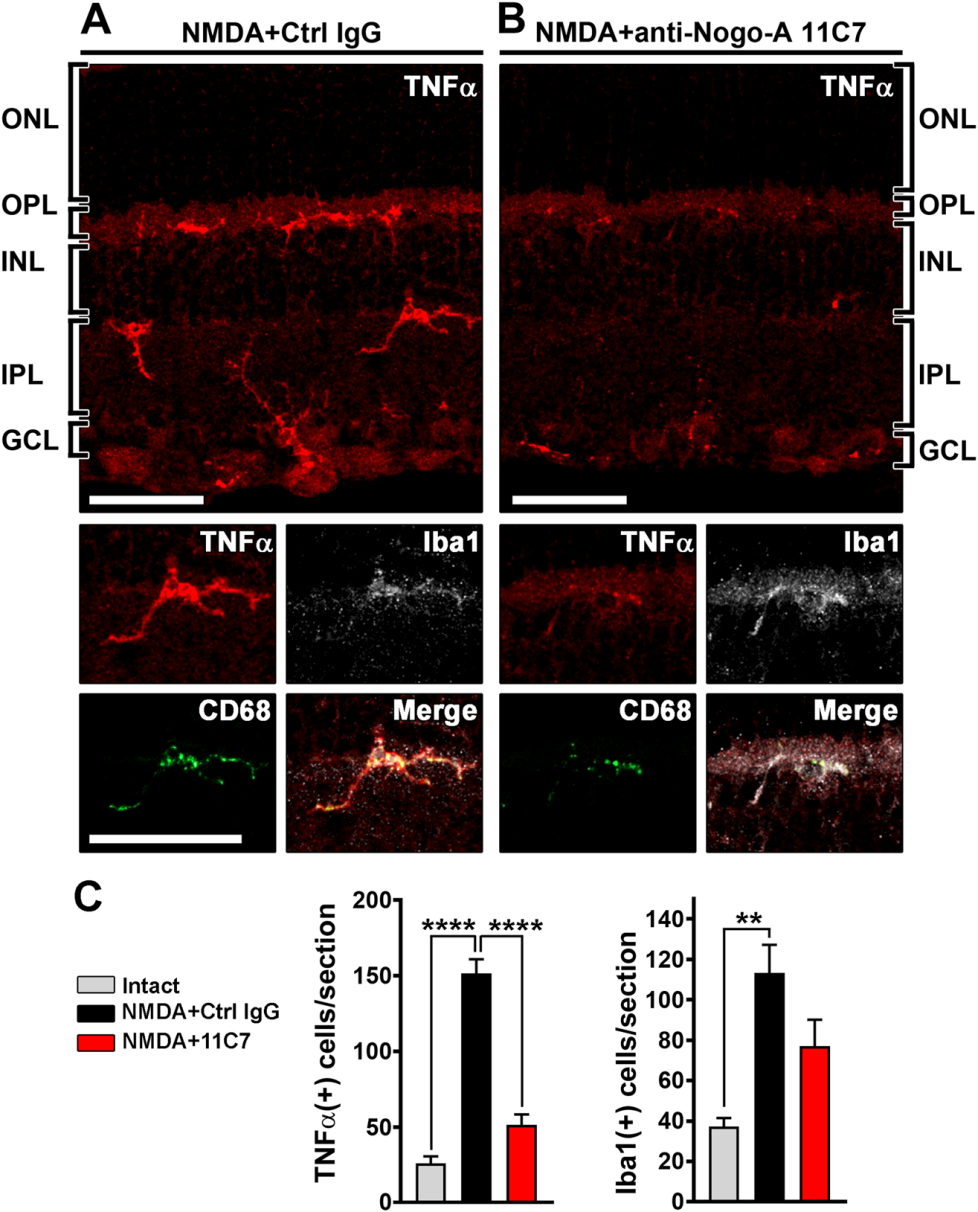
Antibody-mediated Nogo-A blockade decreases the number of cells expressing TNFα without affecting the number of Iba1-positive monocytes. Cells expressing TNFα, Iba1, and CD68 were labeled on cryosections by immunofluorescence in 4 mouse retinae, 24 h after control IgG or 11C7 injection. **a** Retinal sections showed many TNFα-containing cells located in the inner retina, between the outer plexiform layer and the ganglion cell layer 1 day after control IgG injection. A majority of cells was positive for Iba1 and exhibited vesicular staining of the lysosomal protein CD68, indicating their active state. **b** The injection of 11C7 strongly attenuated TNFα and CD68 expression and to a lower extent the intensity of Iba1 in macrophages/microglia. **c** Quantification of the number of cells expressing TNFα and Iba1 suggesting potent and specific effects of 11C7 on TNFα cytokine reduction. Six sections were examined for each retina. Statistics: one-way ANOVA, Dunnett’s post hoc test, **P < 0.01; ****P < 0.0001. Scale bars = 50 μm.

In order to determine the mode of action of 11C7 in retinal function recovery, changes in gene expression were followed by qRT-PCR measurements, after NMDA injection (0.5 nmol) and antibody treatments (Fig. 3a, b). We observed that 11C7 significantly downregulated the expression of genes involved in inflammation and monocyte activation such as those of *Tnf*, *F4/80*, *Csf1*, *Cd68*, 3 days after injury (*P < 0.05; **P < 0.01; Unpaired *t*-test). Strikingly, the mRNA level of *Tnf* was decreased by ~70% and ~80% at 3 and 7 days post injury respectively. *Tnf* and *Sprr1a* mRNA were the only transcripts that showed sustained decreases at 7 days (Fig. 3b). Interestingly, it has been proposed that *Sprr1a* upregulation reflected the RGC response to injury^35,48,49^. The lower level of *Sprr1a* mRNA in 11C7-treated retinae, therefore, suggests weaker RGC stress post-injury, perhaps due to TNFα decrease^50^. To further characterize TNFα protein changes, vitreous samples were analyzed by Western Blotting the day following antibody administration (Fig. 3c). The level of TNFα trimers was reduced by ~70% in eyes receiving 11C7 compared with those injected with control IgG (Fig. 3c, **P < 0.01, unpaired *t*-test). Immunofluorescent stainings allowed to visualize TNFα in Iba1-expressing monocytes (macrophages/microglia) on injured retinal flat-mounts (Fig. 3d). 11C7 markedly decreased the intensity of TNFα signal in NMDA-treated retinae. Quantitatively, the number of TNFα-expressing cells was decreased by ~66% after 11C7 delivery compared with control IgG treatment (Fig. 4a–c). However, the number of Iba1-positive cells did not statistically differ between 11C7 and control IgG treatments (Fig. 4c). According to previous studies, pathological increase of TNFα in the retina can cause delayed and protracted degeneration of axons and oligodendrocytes in the mouse optic nerve^47,51^. However, the examination of olig2-positive oligodendrocytes and Smi32-positive axons did not show differences after NMDA injection and after antibody treatments (Figs. S2, S3). Combined, these results suggest that neutralizing Nogo-A-Delta 20 with a blocking antibody strongly inhibits TNFα expression in microglia/macrophages after retinal injury.

### Nogo-A modulates phosphorylation signalings controlling retinal gliosis

**Fig. 5:**
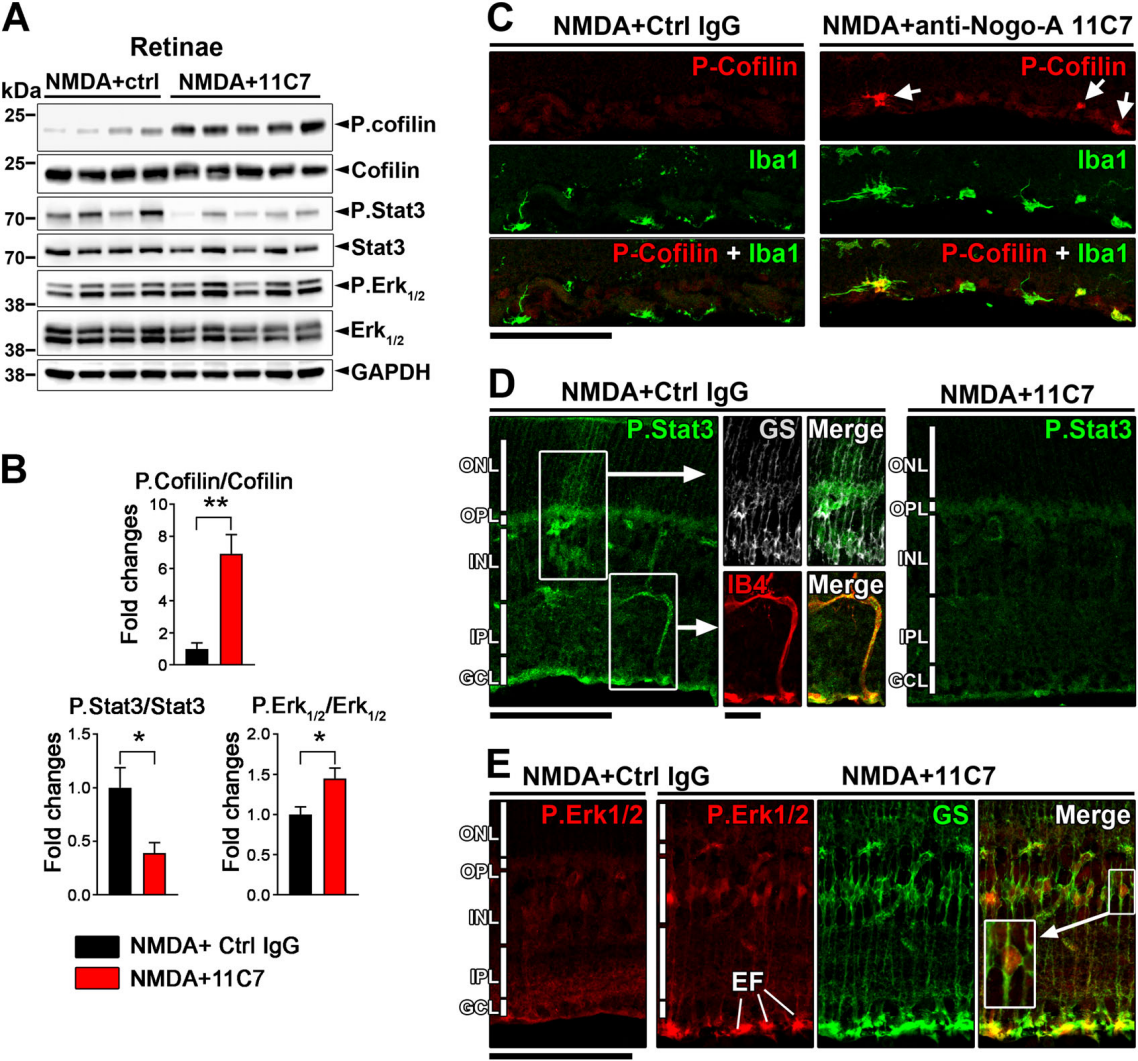
11C7 treatment inhibits inflammatory processes in injured retinal glia. **a**, **b** Western blot analysis of whole retina lysates allowed to detect marked changes in the phosphorylation level of cofilin, P.Stat3 and P.Erk1/2 after NMDA/11C7 injection (*n* = 5 mice) compared with control eye treated with NMDA/control IgG (*n* = 4 mice). In its phosphorylated state, cofilin is inactivated by 11C7. In parallel, P.Stat3 downregulation suggests cytokine signaling reduction. **c** By immunofluorescence on retinal cross sections, P.cofilin upregulation was visualized in microglia/macrophages labeled with Iba1 (arrows). **d** P.Stat3 decrease was from Müller glia positive for glutamine synthetase (GS) and from isolectin B4 (IB4)-labeled blood vessels. **e** Following NMDA and 11C7 treatments, P.Erk1/2 immunoreactivity increased in the nucleus and in the endfeet (EF) of Müller cells identified with cytoplasmic GS. Immunofluorescent stainings were repeated in 3 mice/condition. Scales bars: **c**–**e** = 100 μm, close-up in **d** = 25 μm. ONL, outer nuclear layer; OPL, outer plexiform layer; INL, inner nuclear layer; IPL, inner plexiform layer; GCL, ganglion cell layer. Statistics: *P < 0.05; **P < 0.01; Unpaired *t-*test.

We then sought to know if antibody-mediated Nogo-A blockade modified intracellular phosphorylation cascades involved in retinal cell inflammation and gliosis. To address this, Western blot analysis was focused on cofilin, an actin-severing enzyme whose phosphorylation and inactivation in microglia has been shown to prevent TNFα secretion^52^, and Stat3 and Erk1/2 whose phosphorylation level increases in neuronal and glial cell in response to cytokine stimulation during inflammation^53,54^. The level of phosphorylated/inactive cofilin was dramatically increased by 11C7 compared with control animals receiving IgG (Fig. 5a, b). By immunofluorescence, P.cofilin appeared upregulated in Iba1-expressing microglia/macrophages on retinal sections (Fig. 5c), suggesting that TNFα downregulation may be due to cofilin inactivation in 11C7-treated eyes^52^. In parallel, 11C7 induced P.Stat3 downregulation in Müller glia labeled with GS and in blood vessels stained with isolectin B4 (Fig. 5a, b, d). Stat3 is known to control vimentin and GFAP upregulation in gliotic Müller cells^55^. In the present study, its decrease after 11C7 injection may be responsible for the downregulation of vimentin and, to lower extent *Gfap*, mRNA (Fig. 3a). In addition, P.Erk1/2 upregulation was detected by Western Blotting in 11C7-treated retinal samples (Fig. 5a, b). This increase occurred in Müller cells as shown by the localization of P.Erk1/2 in the nucleus of GS-stained cells (Fig. 5e). P.Erk1/2 elevation may have neuroprotective functions against excitotoxicity in the retina^56^. Together, our results suggest that neutralizing Nogo-A with 11C7 counteracts signaling events controlling gliosis and inflammation.

### Nogo-A is upregulated in the retina of patients with diabetic retinopathy

**Fig. 6:**
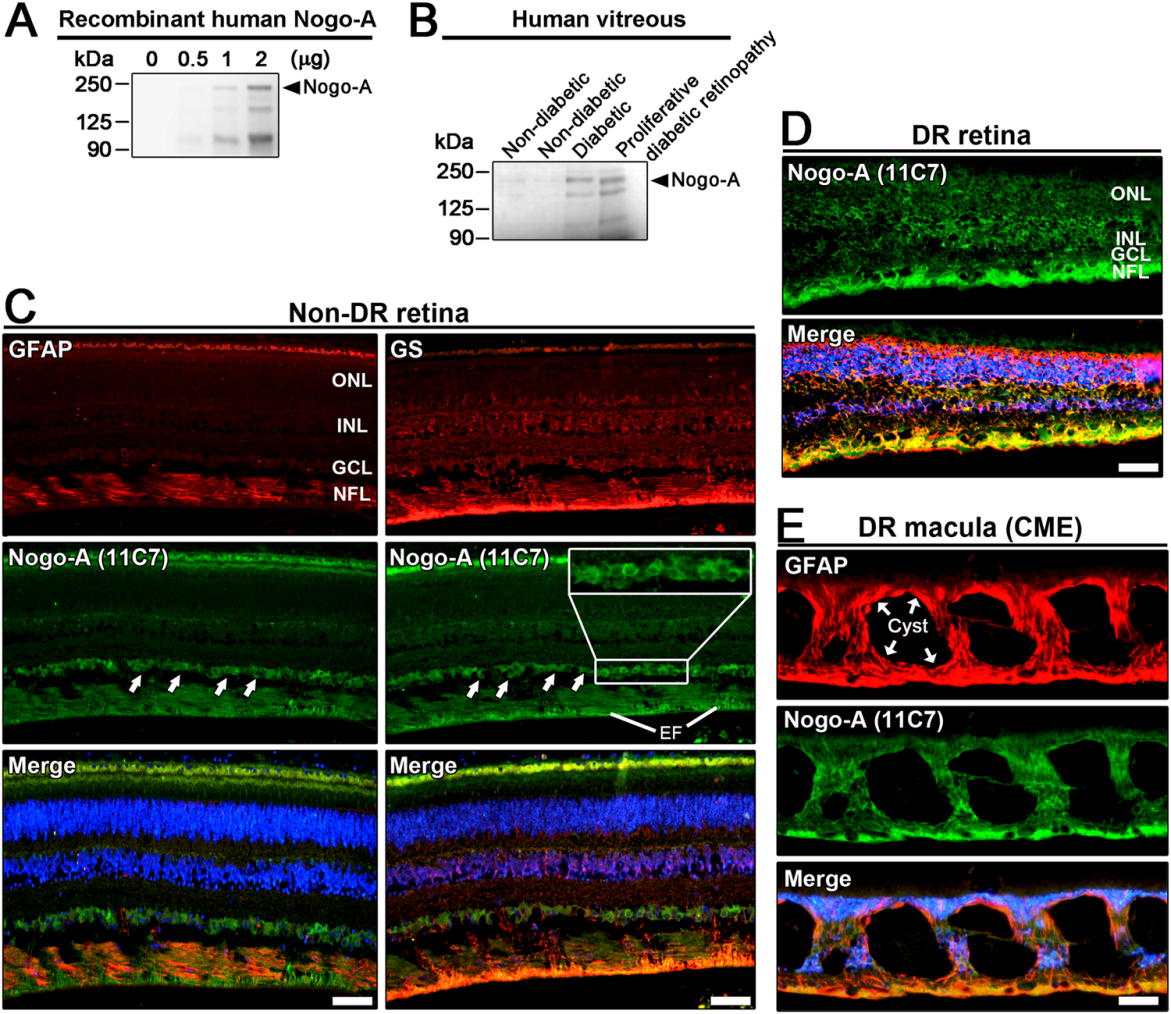
Nogo-A protein is increased in the vitreous and in the retina of patients affected by diabetic retinopathy. **a** Western blot analysis was carried out to detect Nogo-A protein in the vitreous of donors. Anti-Nogo-A antibody allowed to detect recombinant human Nogo-A protein. **b** In the vitreous of non-diabetic patients, Nogo-A signal was very weak in comparison to patients with diabetes and proliferative diabetic retinopathy. **c** Immunofluorescent stainings in human retinal sections allowed to visualize Nogo-A in cell bodies (arrows) of the ganglion cell layer (GCL) and in Müller cell endfeet (EF). Dapi was used to recognize retinal cell layers in blue. To distinguish astrocytes from Müller cell processes in the nerve fiber layer (NFL), GFAP and GS were used respectively as specific cell markers. Without diabetes, astrocytes are the only glia expressing GFAP. They were deprived of Nogo-A staining. In contrast, Nogo-A was colocalized with GS in Müller cell endfeet. **d** In the retina of a diabetic patient, Nogo-A and GS were upregulated in Müller cells whose radial processes span the whole retinal thickness. **e** In the macula of a diabetic retinopathy (DR) patient, severe cystoid macular edema (CME) was observed. Around prominent cysts, Nogo-A and GFAP expression was strongly upregulated in gliotic Müller cells. These observations link Nogo-A expression increase with different stages of diabetic retinopathy. Scale bars = 20 μm.

We then checked if the expression of Nogo-A was increased in the vitreous and in the retina of donors suffering from diabetic retinopathy (DR). At the onset of the disease, the excitotoxic elimination of RGCs and neuroinflammation are thought to mediate visual decline^57–59^. Western blot analysis showed that the level of Nogo-A was increased in the vitreous of patients with diabetes, with or without diabetic retinopathy, compared with samples collected in non-diabetic subjects (Fig. 6a, b). Immunofluorescent observations were then realized on histological sections of retinae from non-diabetic (Fig. 6c) and DR patients (Fig. 6d, e). In the absence of diabetes, Nogo-A was endogenously localized in neuronal cell bodies of the ganglion cell layer and in Müller cell endfeet (Fig. 6c). GFAP-expressing astrocytes were deprived of Nogo-A staining in the nerve fiber layer (NFL) whereas GS-containing Müller cell endfeet exhibited Nogo-A fluorescence (Fig. 6c). In DR retinae, Nogo-A signal was much brighter and colocalized with GS in the endfeet and the radial processes of Müller cell (Fig. 6d). In a severe case of cystoid macular edema (CME), GFAP and Nogo-A expression colocalized in gliotic Müller cells (Fig. 6e). These results suggest that neuronal and glial Nogo-A expression may influence the development of DR in patients.

## Discussion

In this study, we observed the presence of Nogo-A in the mouse vitreous after NMDA-induced retinal injury. The vitreal elevation of Nogo-A occurred during inflammation characterized by TNFα secretion and retinal ganglion cell lysis. Strikingly, delayed injection of a function-blocking antibody directed against Nogo-A-Delta 20 significantly improved visual recovery. However, RGC survival was not influenced by this treatment. The analysis of inflammatory gene expression revealed strong and sustained downregulation of TNFα at the mRNA and protein levels in mice treated with 11C7. A pronounced decrease in TNFα expression was observed by immunofluorescence in microglia/macrophages and may result from cofilin inactivation. In parallel, P.Stat3 downregulation and P.Erk1/2 upregulation in Müller glia suggest that 11C7 attenuates gliosis as well. In general, this study shows that visual function improvement is associated with anti-inflammatory effects of Nogo-A-targeting antibody after retinal injury.

### Retinal excitotoxicity increases vitreal Nogo-A proteins

In healthy conditions, Nogo-A is endogenously expressed in Müller cells and RGCs, but is weakly detectable in the vitreous. Following NMDA-induced injury, the level of Nogo-A proteins was markedly increased in the vitreous. Because Nogo-A is naturally enriched in the endoplasmic reticulum, we propose that vitreal elevation of Nogo-A may stem from RGC lysis, an event that was observed as early as 3 h post-NMDA injection. In contrast, other studies suggested that proteolytic degradation of Nogo-A promoted cell migration^60^ and axonal regeneration^61^. However, in these two last studies, growth activation was obtained in particular conditions, depending on the use of invasive glioblastoma cells^60^ and Schwann cells that stimulate axonal regeneration in the sciatic nerve^61^. It is conceivable that Nogo-A cleavage gives rise to the release of inhibitory fragments contributing to vision loss in the injured eye and that some growth-promoting treatments have the ability to inactivate them by alternative proteolysis. In the present study, vitreal Nogo-A was detected with 11C7, an antibody that selectively binds Delta 20 inhibitory domain^16,62^. In blocking experiments, 11C7 significantly increased visual function, suggesting that vitreal Nogo-A-Delta 20-containing proteins are active in inhibiting retinal neuron recovery. To our knowledge, these findings represent the first hint that active Nogo-A-Delta 20 can be released in biological fluids and diffuse in injured CNS tissues. Therefore, we wonder if the release of Nogo-A proteins also participate in the neurological alterations caused by brain injuries involving excitotoxicity such as cerebral stroke. Indeed, 11C7 can improve function recovery in stroke models^23–25^ in which massive neuronal necrosis produces tremendous inflammation mediated by TNFα^63^. In future studies, it will be important to fully evaluate the therapeutic potential of Nogo-A-neutralizing antibodies by determining if they can modulate neuroinflammation in stroke and spinal injury.

Subsequently to proteolytic cleavage, the fate of Nogo-A-Delta 20-containing proteins has not been addressed in our study. A previous study showed that recombinant Nogo-A-Delta 20 could activate RhoA signaling in neurons upon internalization, thereby inducing growth cone collapse in vitro^64^. Similarly in the retina, endocytosis of Nogo-A-Delta 20 in RGCs and its subsequent axonal transport in vesicles may lead to the inhibition of retinogeniculate terminal plasticity. This mechanism deserves to be tested in the injured visual system. It may allow to explain why the plasticity of RGC terminals is increased in the dorso lateral geniculate nucleus of Nogo-A KO mice after monocular deprivation, for example^36^. A similar mechanism may operate after retinal excitotoxicity.

### Intraocular inhibition of Nogo-A with blocking antibody promotes visual recovery

Delayed blockade of Nogo-A with a single injection of neutralizing antibody was sufficient to promote visual function improvement after retinal injury. This is consistent with our previous results showing that Nogo-A gene deletion allowed better visual recovery in KO mice after NMDA-induced injury^29^. Based on previous observations, a participation of cortical Nogo-A in the restriction of vision recovery remains possible^65^. However, our study reveals that local expression of Nogo-A in the retina contributes to the development of visual deficits after retinal injury.

It is important to underscore the fact that visual improvement was only observed when RGC cell death was ~30% (0.5 nmol NMDA), but not when RGC loss reached ~70% (2 nmol NMDA). Of course, the effects of 11C7 on visual recovery may depend on the level of RGC survival. However, even after the elimination of ~70% of RGCs, a reduced but still measurable OKR was recorded. In this case, additional factors may explain the lack of OKR improvement with 11C7 treatment. In fact, contrary to what has been observed after the injection of 0.5 nmol of NMDA, retinal cell death is not limited to the ganglion cell layer after the delivery of 2 nmol of NMDA, but spread to amacrine cells in the ganglion cell layer and in the inner nuclear layer^29^. In particular, the loss of cholinergic amacrine cells called starburst amacrine cells may cause major OKR decrease. These cells are involved in motion detection and are essential to generate the optokinetic response^66^. Other neurons such as bipolar cells, involved in the production of the ERG b-wave, may be impaired by 2 nmol of NMDA. Indeed, at doses higher than 0.5 nmol, an important functional change associated with NMDA toxicity in the inner nuclear layer was the reduction of ERG b-wave amplitude^29^. In contrast, 0.5 nmol NMDA did not significantly influence ERG b-wave amplitudes compared with PBS injection (data not shown). The decrease in ERG b-wave amplitude noticed after 0.5 nmol NMDA and antibody injections is not the result of retinal damage in the inner nuclear layer but is rather attributable to the injection itself.

In addition, it is important to mention that the effect of 11C7 on visual function enhancement is not restricted to the optokinetic response, a reflexive neck movement induced by the accessory optic system in the brainstem. In a previous study, we found that intravitreally-delivered 11C7 also improved visual cortex response after NMDA-induced retinal excitotoxicity, as shown by the reduction of visual evoked potential latency^29^. Therefore, conscious and non-conscious visual functions may be improved by the blockade of retinal Nogo-A with 11C7 after ocular injury.

### Switch in microglia and Müller glia reaction to injury

Our data showed that the reaction of microglia/macrophages and Müller cell are profoundly modified by 11C7. For microglia/macrophages, the most striking change was the downregulation of TNFα expression in injured retinae. The enhancement of TNFα expression in microglia is a major event controlling retinal excitotoxicity^67^. The elimination or the inhibition of microglia has been shown to prevent NMDA-induced TNFα upregulation in the retina and to protect RGCs from cell death^67^. Given the strong decrease in TNFα expression observed after 11C7 injection, Nogo-A-Delta 20 appears as a new potent modulator of TNFα expression in microglia. Moreover, the decrease of TNFα expression was correlated with cofilin inactivation by phosphorylation. The secretion of TNFα has been shown to be driven by cofilin activation/dephosphorylation in LPS-induced microglia activation^52^. In a similar fashion, 11C7 may reduce the level of TNFα by inactivating cofilin in microglia/macrophages in the retina. Along with TNFα, Sprr1a is the protein whose gene expression was the most downregulated by 11C7 at 7 days post-NMDA delivery. Interestingly, the expression of Sprr1a could be influenced by TNFα in inflammation^50^. It is thus tempting to propose that Nogo-A-Delta 20/P.cofilin/TNFα/Sprr1a may be a stress signaling pathway mediating chronic RGC dysfunction in the injured retina and that may be reversed by 11C7 treatment.

Following retinal injury, TNFα can exert rapid and delayed effects in the development of visual impairments, respectively by regulating RGC death^46^ and by causing oligodendrocyte and optic nerve axon degeneration^51^. Significant loss of axons and oligodendrocytes has been observed in the optic nerve 2 weeks after intravitreal injection of recombinant TNFα, whereas a significant decline in RGC number was only noticed after 2 months^47,51^. Visual function changes have not been followed in these studies. However, we believe that the late visual function improvement that arose at 2 weeks after NMDA injection (12 days after antibody injection) in 11C7-treated mice might result from TNFα downregulation. Unfortunately, in our study, immunofluorescent analysis of optic nerve axons and oligodendrocytes did not allow to find differences between mice receiving 11C7 and control antibody to support this hypothesis. Given the slow progression of axon/oligodendrocyte degeneration initiated by TNFα injections, the histological observations of optic nerves in our study may have been carried out too early (42 days post injury) to detect cellular damage. Alternatively, we propose that 11C7 may improve vision by preserving the integrity of optic nerve oligodendrocyte-axon contacts at the level of the Nodes of Ranvier. The maintenance of the Nodes of Ranvier in the optic nerve is crucial to ensure action potential conduction from the eye to visual brain regions and thus allow visual information integration. For example, when the Nodes of Ranvier are altered in the optic nerve after retinal ischemia, the optokinetic response is markedly impaired^40^. Interestingly, we have previously reported that intravitreal administration of 11C7 reduced visual evoked potential latency in the visual cortex after NMDA-induced excitotoxicity^29^. This observation may result from faster action potential transfer to brain targets. Since intravitreal injection of TNFα can trigger optic nerve demyelination^68^, 11C7-induced TNFα downregulation may lead to a better protection of myelin and the Nodes of Ranvier after excitotoxic injury. This possibility deserves to be addressed in future studies to clarify the mechanisms of action of 11C7 on visual recovery.

### Therapeutic potential of Nogo-A-targeting antibodies in the treatment of retinal diseases

Collectively, the present study and others^28,29^ tend to suggest that Nogo-A may be a relevant molecular target to treat proliferative and non-proliferative diabetic retinopathy. Indeed, Nogo-A expression was dramatically increased in the retina and vitreous of donors with diabetic retinopathy (present study), 2) very similar retinal damages to those produced in our study with NMDA are thought to occur in diabetic retinopathy^5–7,10^. In addition, we have previously demonstrated that 11C7 strongly restricted retinal neovascularization in the model of oxygen-induced retinopathy^28^. Therefore, intravitreal injection of Nogo-A-blocking antibody may reduce neuronal dysfunction and may prevent pathological neovascularization, that are two hallmarks of diabetic retinopathy. In future studies, the effects of Nogo-A antibodies should be assessed in chronic models of diabetic retinopathy involving progressive loss of RGCs and depend on NMDAR activation^2,3^.

## Conclusion

Our results demonstrate that intravitreal injection of anti-Nogo-A antibody can improve visual function and reduce microglia/macrophage-mediated inflammation after retinal injury. These data may be useful to develop new therapeutic approaches for diabetic retinopathy or neurodegenerative diseases with an important inflammatory component.

## Supplementary information

Figure S1

Figure S2

Figure S3

Legends to supplemental figures
