# Supplementary figures and images for: Nogo-A-targeting antibody promotes visual recovery and inhibits neuroinflammation after retinal injury
Source: Cell Death Dis. 2020 Feb 6;11(2):101. doi: 10.1038/s41419-020-2302-x (PMC7005317; doi:10.1038/s41419-020-2302-x)

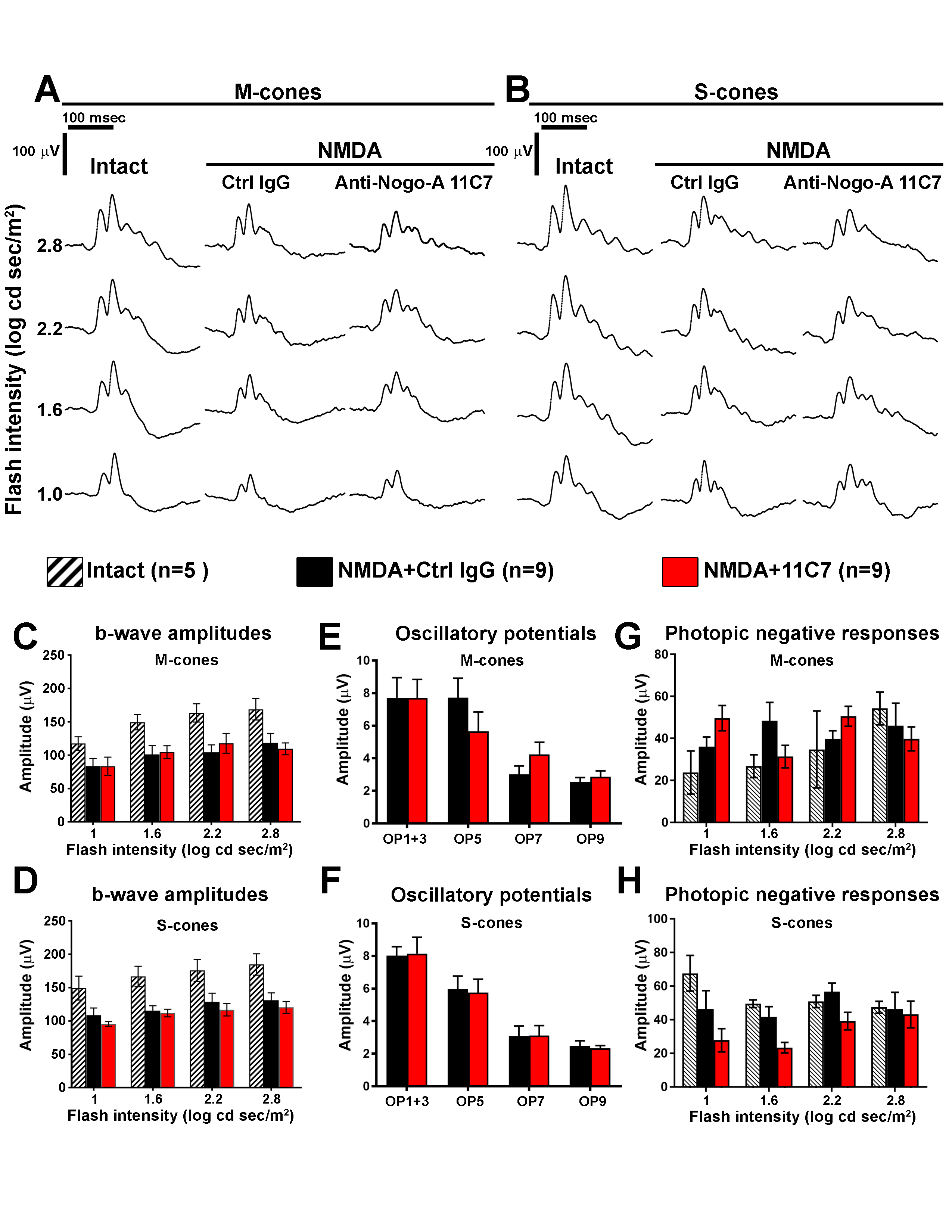

Supplement: Supplementary file 1 — Figure S1 [file 41419_2020_2302_MOESM1_ESM.tif]

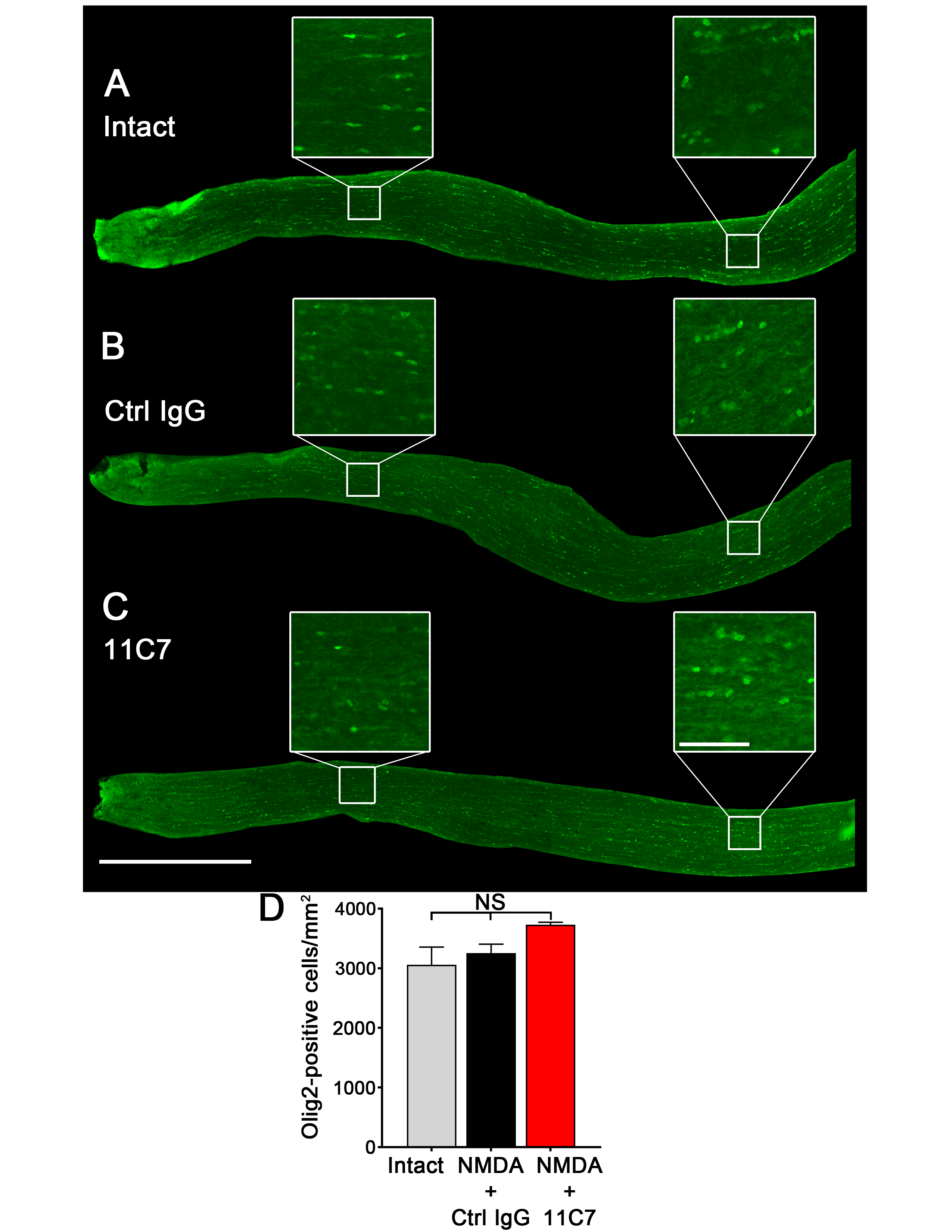

Supplement: Supplementary file 2 — Figure S2 [file 41419_2020_2302_MOESM2_ESM.tif]

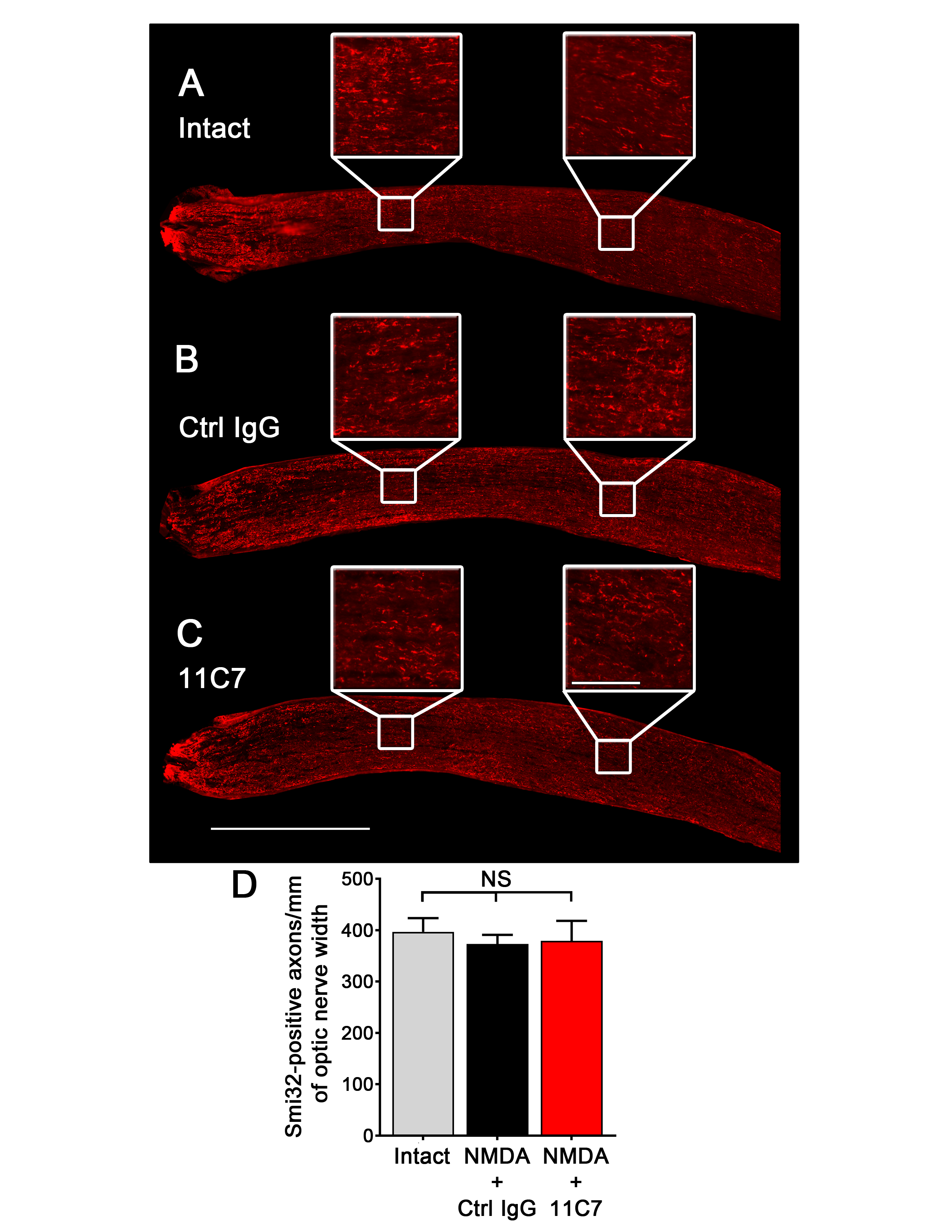

Supplement: Supplementary file 3 — Figure S3 [file 41419_2020_2302_MOESM3_ESM.tif]
