# Supplementary material for: Nogo-A-targeting antibody promotes visual recovery and inhibits neuroinflammation after retinal injury
Source: Cell Death Dis. 2020 Feb 6;11(2):101. doi: 10.1038/s41419-020-2302-x (PMC7005317; doi:10.1038/s41419-020-2302-x)
Supplement: Supplementary file 4 — Legends to supplemental figures [file 41419_2020_2302_MOESM4_ESM.docx]

**Fig. S1. Electroretinogram (ERG) recordings.** **A, B** M-cone- and S-cone-dependent ERG responses were recorded in photopic conditions with light flashes at 504 nm and 365 nm respectively. The amplitude of ERG traces were visibly affected by NMDA (0.5 nmol) in the two mouse groups intravitreally injected with 11C7 (n=9 mice) or control IgG (n=9 mice) compared with intact mice (n= 5 mice). This effect is attributable to intravitreal injection rather than to NMDA-induced toxicity (data not shown) **C, D** Quantitatively, at four intensities of flash stimulation, the ERG b-wave amplitude was similarly reduced by NMDA-induced excitotoxicity in antibody-treated eyes. **E-H** Additional analyses of oscillatory potentials and photopic negative responses, generated with the contribution of RGCs, failed to show an increase after injury and 11C7 administration. Statistics: one-way ANOVA, Dunnett’s post hoc test.

**Fig. S2. Histological analysis of oligodendrocytes in the optic nerve.** Longitudinal optic nerve sections were stained for olig2 to observe oligodendrocyte nuclei. **A** Intact optic nerve oligodendrocytes are arranged in clusters, aligned with the rostro-caudal axis. **B, C** At 42 days post injection, NMDA-induced injury had no obvious effects on the detection of olig2-labeled oligodendrocytes in mice treated with control IgG or 11C7. **D** Quantitatively, the number of oligodendrocytes did not significantly vary between the 3 experimental groups. Five-six optic nerve sections were used in 3 mice used in each experimental group. Statistics: one-way ANOVA, Dunnett’s post hoc test. Scale bar= 500 μm.

**Fig. S3. Histological analysis of RGC axons in the optic nerve.** Longitudinal optic nerve sections were stained for Smi32 to observe alpha RGC axons. **A, B, C** The aspect of Smi32-positive fibers was not distinct between intact nerves (A) and those from NMDA-injected eyes and treated with antibodies (B, C). **D** Quantitatively, the number of axons did not differ between the 3 experimental groups. Statistics: one-way ANOVA, Dunnett’s post hoc test. Scale bar= 500 μm.
